# SNPs in the coding region of the metastasis-inducing gene MACC1 and clinical outcome in colorectal cancer

**DOI:** 10.1186/1476-4598-11-49

**Published:** 2012-07-29

**Authors:** Felicitas Schmid, Susen Burock, Konrad Klockmeier, Peter M Schlag, Ulrike Stein

**Affiliations:** 1 Max-Delbrück-Center for Molecular Medicine, Berlin, Robert-Rössle-Straße 10, 13125, Berlin, Germany; 2 Charité Comprehensive Cancer Center, Berlin, Invalidenstraße 80, 10117, Berlin, Germany; 3 Free University Berlin, Kaiserswerther Str. 16-18, 14195, Berlin, Germany; 4 Experimental and Clinical Research Center, a joint cooperation between the Charité Medical Faculty and the Max-Delbrück-Center for Molecular Medicine, Robert-Rössle-Straße 10, 13125, Berlin, Germany

**Keywords:** Colorectal cancer, Metastasis, MACC1, Single nucleotide polymorphisms

## Abstract

**Background:**

Colorectal cancer is one of the main cancers in the Western world. About 90% of the deaths arise from formation of distant metastasis. The expression of the newly identified gene metastasis associated in colon cancer 1 (MACC1) is a prognostic indicator for colon cancer metastasis. Here, we analyzed for the first time the impact of single nucleotide polymorphisms (SNPs) in the coding region of MACC1 for clinical outcome of colorectal cancer patients. Additionally, we screened met proto-oncogene (Met), the transcriptional target gene of MACC1, for mutations.

**Methods:**

We sequenced the coding exons of MACC1 in 154 colorectal tumors (stages I, II and III) and the crucial exons of Met in 60 colorectal tumors (stages I, II and III). We analyzed the association of MACC1 polymorphisms with clinical data, including metachronous metastasis, UICC stages, tumor invasion, lymph node metastasis and patients’ survival (n = 154, stages I, II and III). Furthermore, we performed biological assays in order to evaluate the functional impact of MACC1 SNPs on the motility of colorectal cancer cells.

**Results:**

We genotyped three MACC1 SNPs in the coding region. Thirteen % of the tumors had the genotype cg (rs4721888, L31V), 48% a ct genotype (rs975263, S515L) and 84% a gc or cc genotype (rs3735615, R804T). We found no association of these SNPs with clinicopathological parameters or with patients’ survival, when analyzing the entire patients’ cohort. An increased risk for a shorter metastasis-free survival of patients with a ct genotype (rs975263) was observed in younger colon cancer patients with stage I or II (P = 0.041, n = 18). In cell culture, MACC1 SNPs did not affect MACC1-induced cell motility and proliferation.

**Conclusion:**

In summary, the identification of coding MACC1 SNPs in primary colorectal tumors does not improve the prediction for metastasis formation or for patients’ survival compared to MACC1 expression analysis alone. The ct genotype (rs975263) might be associated with a reduced survival for younger colon cancer patients in early stages. However, further studies with larger sample sizes are needed.

## Background

Colorectal cancer is the third most common form of cancers in the Western world.^1^ The 5-year-survival rate of colorectal cancer patients with a local tumor is about 90%, whereas only around 10% of the patients survive when distant metastases have formed [1-3].

In an earlier study the new gene metastasis associated in colon cancer 1 (MACC1) was identified by differential display RT-PCR [4]. MACC1 is a prognostic marker for distant metastasis formation and allows the identification of colorectal cancer patients with a high risk for metastatic cancer. Tumors, staged I to III, which developed metachronously metastases, showed a significantly higher MACC1 expression compared to non-metastasizing tumors. The 5-year survival rate for patients with high MACC1 expression in the primary tumors was only 15% compared to 80% for subjects with low MACC1 expression. It was shown that MACC1 is a key regulator of met proto-oncogene (Met) expression [4,5]. The hepatocyte growth factor (HGF)-Met pathway plays a decisive part in epithelial-mesenchymal transition, cell motility, invasiveness, and metastasis [6,7]. Overexpression of MACC1 results in Met expression induction and thereby enhances the activation of the MAPK (mitogen-activated protein kinase)-signaling [4].

Various Met mutations are described that lead to high tumorigenicity. Variants of Met were found in many tumor entities, including colon cancer [8-15]. In contrast to Met mutations, MACC1 mutations in tumors have not yet been studied. In databases, single nucleotide polymorphisms (SNPs) are annotated for MACC1. The three MACC1 SNPs identified in this study (rs4721888, rs975263 and rs3735615) are also annotated in databases. However, their occurrence and frequencies in primary tumors and their association with tumor progression and metastasis are unknown.

Thus, the overriding aim of our study was the evaluation of MACC1 SNPs for prognostication of patients’ survival. For the first time, we analyzed the mutation status of coding MACC1 exons in colorectal tumors and studied the association of MACC1 SNPs with clinicopathological data, including gender, age, tumor stages, lymph node involvement and, in particular, with the development of distant metastases as well as with overall and metastasis-free survival. Furthermore, we studied the functional effect of the identified MACC1 SNPs on the migratory, proliferative or wound healing potential of colorectal cancer cells by *in vitro* assays. In addition, we screened the crucial exons of the proto-oncogene Met in colorectal tumors for mutations.

## Results

### MACC1 SNPs and Met variants in primary colorectal tumors

**Figure 1:**
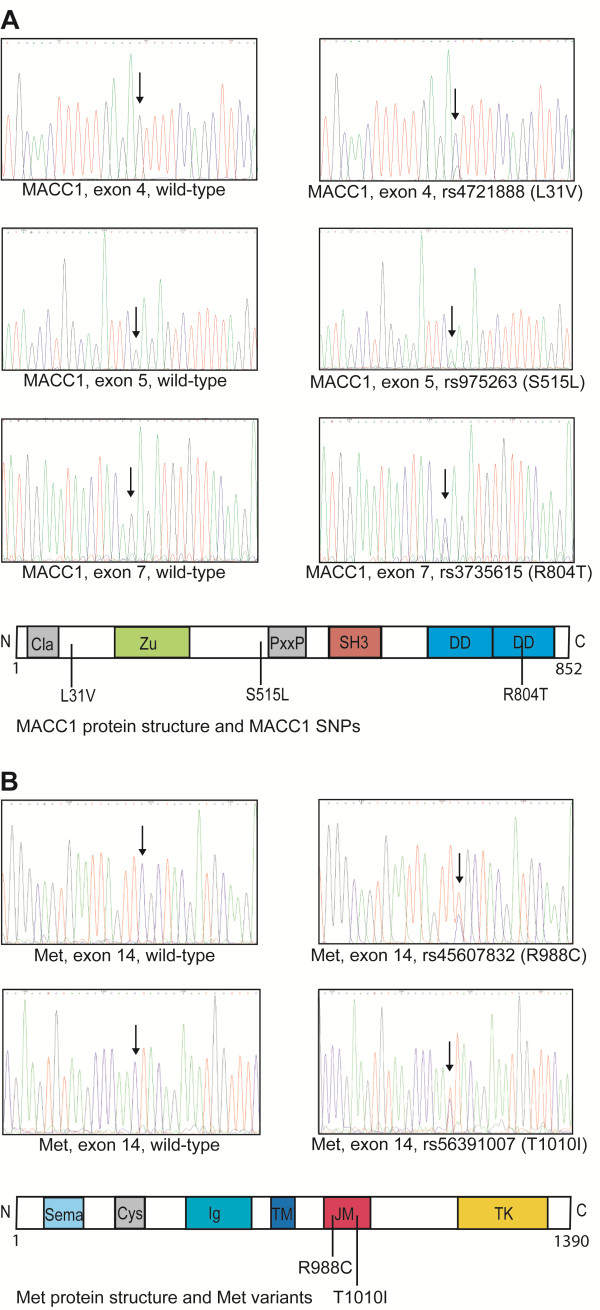
** Genotyped MACC1 SNPs rs4721888, rs975263, rs3735615 and Met variants rs45607832 and rs56391007in primary colorectal tumors. A**) The chromatograms show the wild-type and the MACC1 sequences with the variants. Sites of nucleotide exchanges are indicated by arrow. Location of SNPs in the MACC1 protein structure is marked. Cla: clathrin box, PxxP: proline-rich domain, DD: death domain. **B**) The sequencing chromatograms of two sequences with Met variants R988C and T1010I are shown. Both variants are in the juxtamembrane domain of the Met receptor. Cys: cysteine-rich domain, Ig: immunoglobuline domain, TM: transmembrane domain, TK: tyrosine kinase domain.

We screened the coding exons of MACC1 for mutations and identified the MACC1 genotype cg (31 VL, rs4721888), ct (515 SL, rs975263) and gc or cc (804 RT or 804 TT, rs3735615) in a first panel of 60 colorectal tumors. In order to validate these findings, we screened for these genotypes in a second set of further 94 colorectal tumors and found them as frequent as in the first tumor panel. Thirteen % of the tumors had the genotype cg (31 VL, rs4721888), 48% the ct genotype (515 SL, rs975263,) and 84% a gc or cc genotype (804 RT or 804 TT, rs4721888). Tumors with the variant 31 VL (rs4721888) harbored always the variant 515 SL (rs975263) and variant 804 RT or TT (rs4721888) simultaneously. Furthermore, almost every tumor (97%) with 515 SL (rs975263) also carried the variant 804 RT or 804 TT (rs4721888) (Additional file 1: Table S1). All SNPs were in Hardy-Weinberg-Equilibrium (rs4721888: P = 0.39, rs975263: P = 0.07, rs3735615: P = 1.0). The identified SNPs were in low linkage disequilibrium with r^2^ of 0.05, 0.19 and 0.20 demonstrating that the alleles are randomly distributed. The SNPs are located in the coding MACC1 exons 4 (L31V, rs4721888), 5 (S515L, rs975263) and 7 (R804T, rs3735615) (Figure 1A). In the protein structure the variant L31V appears close to the N-terminus of the MACC1 protein; the polymorphism S515L is located shortly before a putative proline-rich domain, whereas R804T occurs in a putative second death domain of the MACC1 protein (Figure 1A).

Furthermore, we studied the coding exons 14 to 19 of one of the transcriptional targets of MACC1, the proto-oncogene Met, in the first 60 colorectal tumors. For this mutation analysis we chose the juxtamembrane and the kinase domain because most of the already described mutations were found in these domains and were described to affect the function of this receptor tyrosine kinase [8,15]. We found only two tumors with Met mutations: in one tumor the variant R988C (rs45607832) and in a second tumor the variant T1010I (rs56391007) (Figure 1B). Both variants occurred in the juxtamembrane domain (exon 14) of the receptor (Figure 1B). Because of the low minor allele frequency no further tumors were sequenced.

### MACC1 SNPs have no impact on MACC1 expression

**Figure 2:**
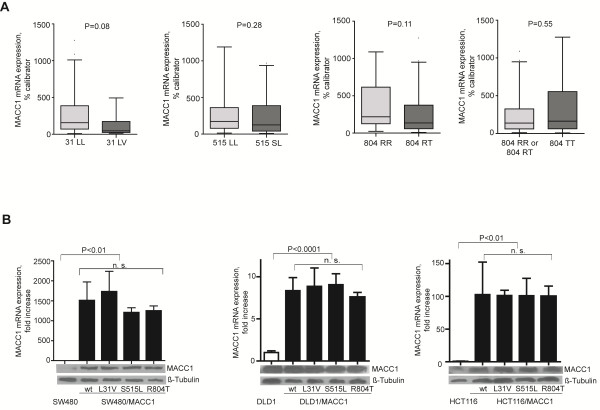
** MACC1 SNPs L31V, S515L, and R804T and MACC1 expression in colorectal cells and tumors A).** MACC1 mRNA expression was determined in 60 colorectal tumors by qRT-PCR. Box plots show the mRNA expression compared to MACC1 genotypes. SNPs have no effect on the MACC1 mRNA expression in these colorectal tumors. **B**) SW480, DLD1, HCT116 colorectal cancer cells were transfected with pcDNA3.1/MACC1/wt, pcDNA3.1/MACC1/L31V, pcDNA3.1/MACC1/S515L and pcDNA3.1/MACC1/R804T. The MACC1 mRNA expression in these cell clones was measured by qRT-PCR and normalized to the MACC1 mRNA expression of parental cells. Additionally, MACC1 protein expression was determined. β-tubulin was used as a loading control. SNPs have no effect on the MACC1 mRNA and protein expression in SW480, DLD1 and HCT116 colorectal cancer cells.

We analyzed the MACC1 mRNA expression in the set of 60 colorectal tumors (Figure 2A). We compared each SNP to the mRNA expression of MACC1 in the tumors and did not find an association of the SNPs with MACC1 expression (L31V: P = 0.08, S515L: P = 0.28, R804T: P = 0.11, R804T homozygous: P = 0.55).

Moreover, we evaluated the impact of the identified SNPs on the expression of MACC1 in cell culture. We used the colorectal cancer cell lines SW480, DLD1 and HCT116. We introduced SNPs into the wild-type MACC1 sequence by site-directed mutagenesis, cloned the plasmids pcDNA3.1/MACC1/wt, pcDNA3.1/MACC1/L31V, pcDNA3.1/MACC1/S515L, pcDNA3.1/MACC1/R804T, and transfected SW480, DLD1 and HCT116 cells with these constructs. MACC1-transfected cells had an increased MACC1 mRNA (P < 0.01, P < 0.0001, P < 0.01) and protein expression levels compared to parental cells. The identified SNPs, however, did not influence the MACC1 mRNA (P = 0.37, P = 0.54, P = 0.99) or protein expression levels compared to SW480/MACC1/wt, DLD1/MACC1/wt or HCT116/MACC1/wt cells (Figure 2B).

### MACC1 SNPs do not affect motility and proliferation of colorectal cancer cells

**Figure 3:**
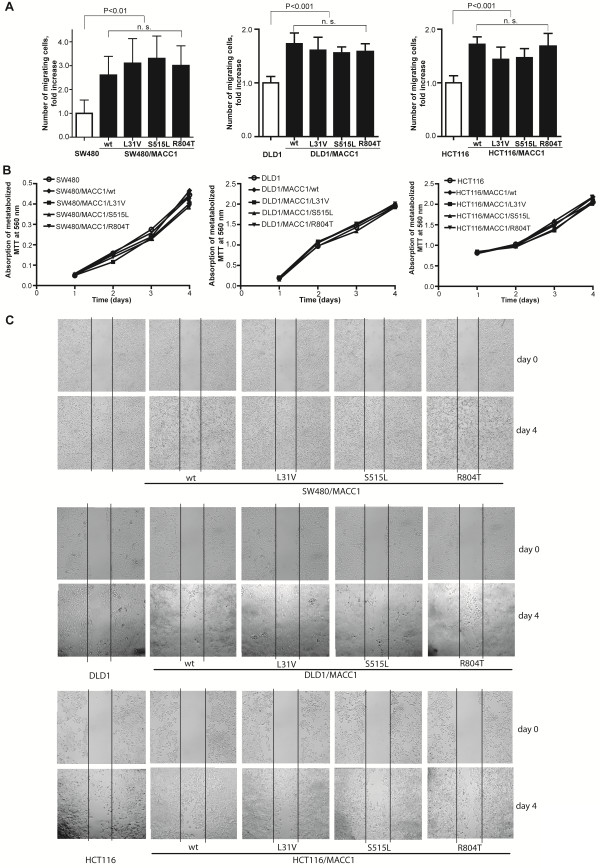
** MACC1 SNPs L31V, S515L, and R804T, cell motility, and proliferation in colorectal cancer cells. A**) SW480, DLD1 and HCT116 colorectal cancer cells were transfected with pcDNA3.1/MACC1/wt, pcDNA3.1/MACC1/L31V, pcDNA3.1/MACC1/S515L and pcDNA3.1/MACC1/R804T. Cell migration was analyzed using Boyden chamber assay. SNPs have no effect on the migratory abilities of the cells. **B**) The proliferation of SW480, DLD1, HCT116 and transfected cells was analyzed by MTT assay. SNPs do not affect the proliferative potential of the cells. **C**) For the wound healing assay SW480, DLD1, HCT116 and transfected cells were seeded and scratched. The wound healing area was documented until day 4. Cells with MACC1 SNPs show the same abilities to close the wound compared to cells with wild-type MACC1.

In order to evaluate the impact of MACC1 SNPs on the biological functions of MACC1 we performed *in vitro* assays. SW480, DLD1 and HCT116 cells transfected with the MACC1 constructs pcDNA3.1/MACC1/wt, pcDNA3.1/MACC1/L31V, pcDNA3.1/MACC1/S515L, pcDNA3.1/MACC1/R804T were used for these biological assays. We evaluated the migratory impact of SW480, DLD1 and HCT116 cells and transfectants thereof (Figure 3A). We previously demonstrated increased SW480 cell migration ability of wild-type MACC1 transfectants compared to parental SW480 cells [4]. In this study, we also observed an about 3-fold increased cell migration of SW480/MACC1/wt, a 1.7-fold increased cell migration of DLD1/MACC1/wt and of HCT116/MACC1/wt cells compared to the parental cell lines (P < 0.01, P < 0.001, P < 0.001). However, this MACC1-induced cell migration was comparable in all MACC1-transfected cell clones independent of the introduced SNPs. Thus, the MACC1 SNPs L31V, S515L, and R804T have no impact on the migratory ability of colorectal cancer cells (P = 0.77, P = 0.12, P = 0.27) (Figure 3A). 

Moreover, we studied the proliferative potential of the transfected cells. Cells with one of the constructs pcDNA3.1/MACC1/L31V, pcDNA3.1/MACC1/S515L or pcDNA3.1/MACC1/R804T showed the same ability for proliferation as cells with the wild-type construct. Thus, none of the SNPs affects the proliferative potential of these colorectal cancer cells (Figure 3B).

Furthermore, we analyzed the wound healing ability of these cells. MACC1 promotes the ability of cells to close a wound [4]. We confirmed that SW480/MACC1/wt, DLD1/MACC1/wt and HCT116/MACC1/wt cells do close the wound area faster than parental cells, but transfectants harboring the MACC1 SNPs L31V, S515L, and R804T did not demonstrate an altered potential for directed migration (Figure 3C).

### MACC1 SNPs and association with metastasis or clinicopathological parameters

We analyzed if variants 31 LV, 515 SL or 804 RT or 804 TT are associated with clinicopathological factors of all 154 patients (Table 1). We compared each polymorphism to the formation of metachronous metastasis of the subjects, to age and gender of the patients, and to clinical parameters including UICC-stages, tumor or lymph node infiltration. None of the parameters is significantly associated with any of the identified polymorphisms (for details see Table 1).

**Table 1:** Association of MACC1 SNPs with clinicopathological factors

**rs4721888 (L31V)**						
**Parameters**	**Leu/Leu (ctc/ctc) (%)**		**Leu/Val (ctc/gtc) (%)**	**P-value**	**OR**	**95% CI**
All tumors, n = 154	134 (87%)		20 (13%)			
Metachronous metastasis,n = 29	27 (93%)		2 (7%)	0.28	0.44	0.10 - 2.02
Gender male, n = 79	68 (86%)		11 (14%)	0.72	1.19	0.46 - 3.05
Age > 66.32 years (median),n = 77	67 (87%)		10 (13%)	1.00	1.00	0.39 – 2.56
pT3 and pT4, n = 118	102 (86%)		16 (14%)	0.70	1.26	0.39 - 4.03
N1 to N3, n = 53	43 (81%)		10 (19%)	0.12	2.12	0.82 - 5.47
UICC stage I, n = 26	24 (92%)		2 (8%)	0.38	0.51	0.11 - 2.34
UICC stage II, n = 74	66 (89%)		8 (11%)	0.44	0.69	0.26 - 1.79
UICC stage III, n = 54	44 (81%)		10 (19%)	0.13	2.05	0.79 - 5.28

**rs975263 (S515L)**						
**Parameters**	**Ser/Ser (tcg/tcg) (%)**		**Ser/Leu (tcg/ttg) (%)**	**P-value**	**OR**	**95% CI**
All tumors, n = 154	80 (52%)		74 (48%)			
Metachronous metastasis,n = 29	12 (41%)		17 (59%)	0.21	1.69	0.75 – 3.83
Gender male, n = 79	40 (51%)		39 (49%)	0.74	1.11	0.59 - 2.10
Age > 66.32 years (median),n = 77	40 (52%)		37 (48%)	1.00	1.00	0.53 – 1.88
pT3 and pT4, n = 118	63 (53%)		55 (47%)	0.52	0.78	0.37 - 1.65
N1 to N3, n = 53	26 (49%)		27 (51%)	0.60	1.19	0.61 - 2.32
UICC stage I, n = 26	14 (54%)		12 (46%)	0.83	0.91	0.39 - 2.13
UICC stage II, n = 74	39 (53%)		35 (47%)	0.86	0.94	0.50 - 1.78
UICC stage III, n = 54	27 (50%)		27 (50%)	0.72	1.13	0.58 - 2.19

**rs3735615 (R804T)**				**without R804T compared to with R804T**		
**Parameters**	**Arg/Arg (aga/aga) (%)**	**Arg/Thr (aga/aca) (%)**	**Thr/Thr (aca/aca) (%)**	**P-value**	**OR**	**95% CI**
All tumors, n = 154	25 (16%)	74 (48%)	55 (36%)			
Metachronous metastasis,n = 29	5 (17%)	15 (52%)	9 (31%)	0.95	0.96	0.33 - 2.80
Gender male, n = 79	11 (14%)	37 (47%)	31 (39%)	0.43	1.42	0.60 - 3.36
Age > 66.32 years (median),n = 77	12 (16%)	39 (51%)	26 (34%)	0.83	1.10	0.47 - 2.59
pT3 and pT4, n = 118	21 (18%)	53 (45%)	44 (37%)	0.34	0.58	0.18 - 1.81
N1 to N3, n = 53	9 (17%)	27 (51%)	17 (32%)	0.86	0.92	0.38 - 2.25
UICC stage I, n = 26	4 (15%)	14 (54%)	8 (31%)	0.90	1.08	0.34 - 3.46
UICC stage II, n = 74	12 (16%)	32 (43%)	30 (41%)	1.00	1.00	0.43 - 2.36
UICC stage III, n = 54	9 (17%)	28 (52%)	17 (32%)	0.91	0.95	0.39 - 2.33

SNPs are not associated with clinicopathological data like metachronous development of distant metastases, gender, age (median: 66.32 years), tumor stages (pT3, pT4), lymph node status (N1 to N3: with lymph node metastases) and UICC stages. Ser: serine, Leu: leucine, Arg: arginine, Thr: threonine. P-values were determined by using Chi-Square test and odds ratios (OR) with 95% confidence intervals (95% CI) were calculated.

### MACC1 SNPs and association with overall survival (OS) and metastasis-free survival (MFS) in all colorectal cancer patients

We analyzed OS and MFS of all 154 colorectal cancer patients with variants 31 LV, 515 SL, 804 RT or 804 TT. We did not find an association with OS or MFS of all patients with any of the identified MACC1 SNPs (OS: P = 0.77, HR = 1.14, 95% CI = 0.49-2.66; MFS: P = 0.71, HR = 1.21, 95% CI = 0.44-3.38) (Figure 4.1A). When analyzing each SNP separately, no association of any of the SNPs with OS or MFS was found: 31 LV (OS: P = 0.79, HR = 0.88, 95% CI = 0.36-2.18, MFS: P = 0.40, HR = 1.62, 95%CI = 0.53-4.97, Figure 4.1B); 515 SL (OS: P = 0.68, HR = 0.88, 95% CI = 0.48-1.60, MFS: P = 0.21, HR = 0.63, 95% CI = 0.30-1.30, Figure 4.1 C), patients with variant 804 RT or 804 TT had no decreased survival time (804 RT: OS: P = 0.98, HR = 0.99, 95% CI = 0.44-2.22, MFS: P = 0.91, HR = 1.06, 95% CI = 0.40-2.82, Figure 4.1 D; 804 TT: OS: P = 0.81, HR = 1.08, 95% CI = 0.58 - 2.01, MFS: P = 0.62, HR = 1.22, 95% CI = 0.57-2.60, Figure 4.1 E).

**Figure 4:**
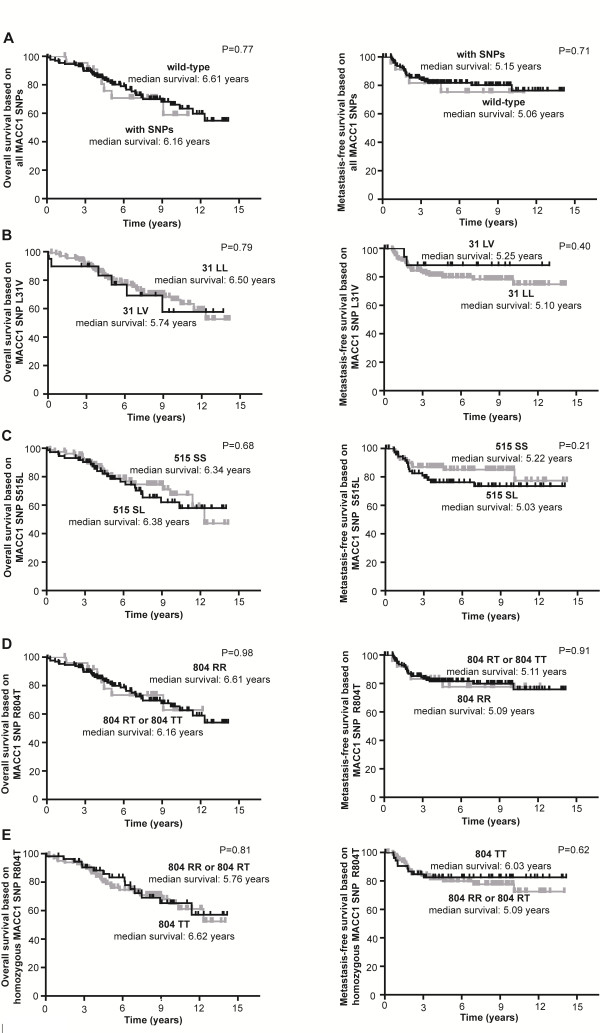
** MACC1 SNPs rs4721888, rs975263, rs3735615 and colorectal cancer patients’ survival.** OS and MFS of 154 colorectal cancer patients was analyzed with respect to **A**) SNPs rs4721888, rs975263, rs3735615 vs. no variants, **B**) variant 31 LL vs. 31 LV, **C**) 515 SS vs. 515 SL, **D**) 804 RR vs. 804 RT/TT, **E**) 804 RR/RT vs. 804 TT. Significant differences of survival times in comparison to MACC1 SNPs were not found. Survival rates were assessed using the Kaplan-Meier method and differences between groups were assessed using the Log-Rank tests.

Combinations of SNPs, e.g. patients with the variant allele 31 LV and 515 SL, with 31 LV and 804 RT/TT, or with 515 SL and 804 RT/TT, compared to patients without any SNP or with only one SNP did not improve survival prognosis.

### Younger colon cancer patients of early stages with a genotype 515 SL have a reduced MFS

**Figure 5:**
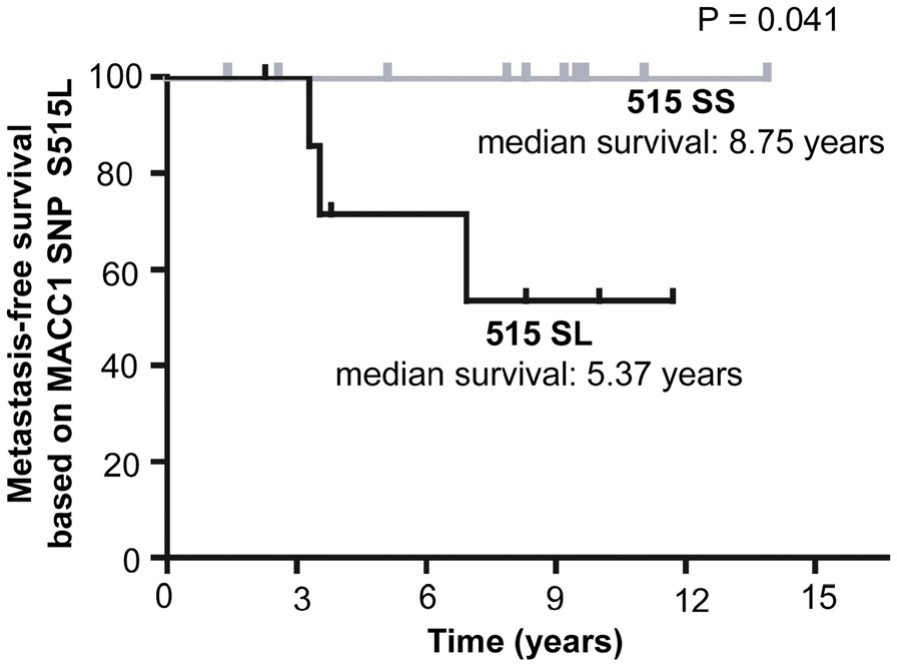
** MFS of younger patients with early staged colon cancer with MACC1 SNP rs975263.** 515 SL was associated with a reduced MFS time of younger colon cancer patients (<60 years, time at diagnosis) in stage I or II (P = 0.041, n = 18). P-value was calculated by Log-Rank test.

When we determined the MFS time of a small group (n = 18) of the 154 colorectal cancer patients we found an association of the MFS time with the genotype 515 SL (Figure 5). Patients with colon cancer in UICC-stage I or II who are younger than 60 years (age at diagnosis) had a reduced MFS time when they harbored the T-allele compared to patients with the C-allele (P = 0.041, HR = 0.09, 95% CI = 0.01-0.91).

## Discussion

The metastasis-inducing gene MACC1 is a newly identified gene and its expression is a prognostic indicator for colorectal cancer metastasis [4]. In this study, we identified the SNPs rs4721888, rs975263, rs3735615 in the coding region of MACC1 in primary colorectal tumors. We analyzed the association of the MACC1 SNPs with clinical parameters such as age, gender as well as with metachronous metastasis, UICC-stages, tumor and lymph node infiltration. However, none of the identified variants 31 LV, 515 SL, and 804 RT or 804 TT was associated with one of these parameters. Furthermore, the identified SNPs were neither associated with OS nor with MFS in the entire cohort of colorectal cancer patients analyzed. However, colon cancer patients classified to UICC-stage I or II and younger than 60 years old with a 515 SL variant had a decreased MFS time. Thus, the T-allele of this SNP might be associated with the survival of younger patients with an early staged colon tumor. Nevertheless, our analyzed subgroup is very small and validation by comprehensive cohorts is necessary.

We identified three different MACC1 SNPs and two Met variants in the colorectal tumors that are already annotated in the NCBI SNP database. The occurrence and frequencies of MACC1 SNPs in primary tumors of colorectal cancer patients were unknown but their frequencies in different non-cancer populations can be found in SNP databases. Only two tumors showed variants of the proto-oncogene Met. Other studies confirmed that Met mutations are rare events [11,16]. Tyner and colleagues reported that the variant R988C occurs only in 1% (95% CI = 0-5%) and T1010I in 0% (95% CI = 0-3%) of 109 colorectal tumors [11]. In our analyzed tumors both Met variants appear in only 1.6% of all tumors. The effects of the Met variants are controversially discussed. Schmidt et al. reported that T1010I does not result in a constitutive phosphorylation of Met in NIH3T3 cells [15]. Lee et al. confirmed the conclusion, but showed that mice tumors with this Met variant grow slightly faster [12]. Finally, Tyner et al. described no difference in the transforming capacity or in the phosphorylation status of the variants compared to the wild-type receptor [11]. Due to the few Met variants in our tumor panel a correlation to clinical data was not possible.

It was shown that various SNPs of cancer-related genes are associated with a higher risk or a faster progression of cancers [17-19]. Examples of activating mutations are the missense mutations in codon 12 or 13 of the KRAS genes that lead to conformational changes in the KRAS protein [20]. In SNP databases 55 missense SNPs in the coding region of MACC1 are annotated. All three identified MACC1 SNPs in this study are missense alterations. SNP L31V leads to an amino acid exchange from leucine to valine. An influence on the protein structure is disputable because both amino acids belong to the group of nonpolar amino acids. SNP S515L results in a substitution of serine for leucine. Here, a polar charged amino acid is replaced by a nonpolar one and this may result in a loss of a possible phosphorylation site. SNP R804T exchanges the amino acid sequence from arginine to threonine that potentially can act as phosphorylation site. Thus, an effect on the protein function could be possible. We used SIFT and Polyphen software tools for the estimation of functional impact [21,22]. Both programs predict that the 31 VL and 515 SL variants are probably benign, whereas 804 RT could be damaging. R804T lies in a conserved domain and might have therefore a decisive role. SNPs L31V and S515L are not in predicted domains of the protein structure, whereas R804T can be found in a putative death domain. So far, the MACC1 protein domain architecture was exclusively studied by bioinformatical approaches [23]. Further efforts are required to elucidate the MACC1 protein structure in order to evaluate the importance of the identified SNPs. We analyzed the possible effect of these MACC1 SNPs on the protein function by biological *in vitro* assays. First, we showed that the SNPs do not change the expression level of MACC1. We then performed migration and proliferation assays but did not observe an influence of the SNPs on the behavior of the colorectal cancer cells. The MACC1 variant 804 RT or 804 TT which was predicted as possibly damaging did not have a functional impact on cell migration and proliferation studies.

This study has some limitations. First, as already mentioned, the MACC1 SNP analysis should also be carried out with a larger cohort of patients. Further efforts should be made in order to evaluate if the MACC1 SNPs are germline or somatic polymorphisms and if they occur in other tumor entities. Moreover, other regions of the MACC1 gene that are more likely related to its expression, such as the promoter region or the 3’UTR where possible miRNAs could bind, should be explored.

## Conclusion

Taken together, the analysis of the coding MACC1 variants 31 LV, 515 SL, 804 RT or 804 TT in primary colorectal tumors does not improve the prediction for metastasis formation or for patients’ survival compared to MACC1 expression analysis alone. Thus, a general screening for these MACC1 SNPs in colorectal cancer patients is not recommended. This first study suggests that the MACC1 SNP S515L should be validated in a further study in order to prove a possible prognostic value for this SNP.

## Methods

### Patients

In total, 154 patients with pathologically confirmed primary colorectal tumors (adenocarcinomas) with UICC-stages I, II and III were enrolled in this study. A comprehensive overview of patients’ data is shown in Table 2. Primary colorectal tumors were obtained from all patients with informed written consent (approved by the local ethics committee of the Charité, Berlin). Tumor staging and typing was performed according to UICC and WHO guidelines. The subjects were previously untreated, did not have a history of familial colorectal cancer, did not suffer from a second tumor of the same or a different entity, and underwent surgical R0 resection. Twenty-nine subjects developed metachronously distant metastases. The follow-up data of all patients was documented for more than 5 years and up to 14 years (with a median follow-up of 6.3 years) after diagnosis. For patients with MACC1 variant 31 LV the median follow-up time was 5.7 years, for 515 SL 6.4 years and for 804 RT or 804 TT 6.2 years. Overall survival was calculated from date of histopathological diagnosis to the date of death from any cause; patients known to be alive at last contact were censored. Metastasis-free survival was determined from the date of histopathological diagnosis to the time on which the development of distant metastasis was observed.

**Table 2:** Characteristics of colorectal cancer patients

**Characteristics**	**n (%)**
Ethnicity	
European	154 (100%)
Gender	
Male	79 (51%)
Female	75 (49%)
Age at diagnosis (years)	
Median ± SD: 66.32 ±10.44	
< 60	40 (26%)
60-70	57 (37%)
> 70	57 (37%)
Localisation	
Colon	105 (68%)
Rectum	49 (32%)
Metachronous metastasis	
With	29 (19%)
Without	125 (81%)
UICC stage	
I	26 (17%)
II	74 (48%)
III	54 (35%)
pT status	
pT1 + 2	36 (23%)
pT3 + 4	118 (77%)
pN status	
Negative	101 (66%)
Positive	53 (34%)

SD indicates the standard deviation. Tumors were classified according to the guidelines of the nternational Union Against Cancer (UICC) staging system. International Union Against Cancer (UICC) staging system.

### Microdissection and isolation of DNA and RNA

Serial cryosections of each tissue were evaluated by a pathologist. The tumor cell populations were microdissected and the DNA was isolated with the QIAamp Minikit (Qiagen) according to the manufacturer’s protocol. Total RNA was extracted by TRIzol/Chloroform (Invitrogen).

### Polymerase chain reaction (PCR) and sequencing

All coding MACC1 exons 4 to 7 and the Met exons 14 to 19 were amplified by PCR. PCR was performed with proof-reading Pfu DNA polymerase (Fermentas) in a final volume of 50 μl. Reactions contained 10x Pfu buffer with 2.5 mM MgSO_4_ (Fermentas), 0.2 mM of each dNTP (Applied Biosystems), 0.3 μM of each primer, 25 ng DNA and 2.5 u Pfu DNA polymerase. The sequences of primers are listed in Table 3. Purification and sequencing of PCR products were done by AGOVA GmbH (Berlin).

**Table 3:** Primers and probes used for PCR and qRT-PCR

**Gene**	**Primer/Probe**	**Sequence 5’-3’**
MACC1, exon 4	Forward primer	atctagtcgagtatcctaccag
	Reverse primer	cagaggtagaccttcaacaattat
MACC1, exon 5.1	Forward primer	cttgattgtaactcacagtgcc
	Reverse primer	gaggttgcctaacatgatttcc
MACC1, exon 5.2	Forward primer	gaattccaagaggtgtctctaag
	Reverse primer	cttcacctgcttccaactgc
MACC1, exon 5.3	Forward primer	ggacacaattatatgccaggac
	Reverse primer	gcagtgtacaagtccaatcttac
MACC1, exon 5.4	Forward primer	ggacacaattatatgccaggac
	Reverse primer	gcagtgtacaagtccaatcttac
MACC1, exon 5.5	Forward primer	gcagtgctaagacaaagcaag
	Reverse primer	catttctcctctcacatggttcag
MACC1, exon 6	Forward primer	ctctggcttagttatgtctactg
	Reverse primer	gtgaatccgtgaatgtggtatg
MACC1, exon 7	Forward primer	gtccatgtgtaattggtattccg
	Reverse primer	tctgagattctttctttcctacac
Met, exon 14	Forward primer	gtcgattcttgtgtgctgtctt
	Reverse primer	cagaggtaaatacttcctttagg
Met, exon 15	Forward primer	gctaccactgcttccattcttaaggac
	Reverse primer	ttgcttccatgcacaagggcaaatcc
Met, exon 16	Forward primer	gcttatatccttgggtgaaatgtgttgcatc
	Reverse primer	atgagggctctgagggatcatttcag
Met, exon 17	Forward primer	aaccctcaggacaagatgctaa
	Reverse primer	ggtgcatttgaatgatgctaacat
Met, exon 18	Forward primer	aggcttgagccattaagaccaa
	Reverse primer	ccagggcttacacatcgattta
Met, exon 19	Forward primer	gaggccagatgaaatacttcct
	Reverse primer	atgaagaaaactggaattggtggt
MACC1, SNP L31V	Forward primer	gaagctggaaaagtctcaaaaagtt
	Reverse primer	aactttttgagacttttccagcttc
MACC1, SNP S515L	Forward primer	taaaaagactcttgaatctgccagg
	Reverse primer	cctggcagattcaagagtcttttta
MACC1, SNP R804T	Forward primer	gaaataactacacagatgtgttaca
	Reverse primer	tgtaacacatctgtgtagttatttc
MACC1, qRT-PCR	Forward primer	ttcttttgattcctccggtga
	Reverse primer	actctgatgggcatgtgctg
	FITC-probe	gcagacttcctcaagaaattctggaagatcta-FITC
	LCRed640	LCRed640-agtgtttcagaacttctggacattttagacga

### Quantitative reverse transcription–polymerase chain reaction (qRT-PCR)

QRT-PCR was carried out using the LightCycler480 (Roche) as described previously [4]. QRT-PCR was performed with the FastStart DNA Master HybProbe Kit (Roche) according to the manufacturer’s instructions. The primers and probes are listed in Table 3. In addition, cDNA quantification of the housekeeping gene glucose-6-phosphate dehydrogenase (Roche) was performed. For each qRT-PCR reaction a mean of duplicates was calculated and normalized to the respective mean of the housekeeping gene cDNA.

### Site-directed mutagenesis

The MACC1 SNPs L31V, S515L and R804T were introduced into the previously described plasmid pcDNA3.1D/MACC1-V5-His by site-directed mutagenesis (QuikChange XL Site-Directed Mutagenesis Kit, Stratagene) [4]. The primers are summarized in Table 3. The constructs pcDNA3.1/MACC1/wt, pcDNA3.1/MACC1/L31V, pcDNA3.1/MACC1/S515L and pcDNA3.1/MACC1/R804T were used for transfection of colorectal cancer cells.

### Transfection of SW480, DLD1 and HCT116 cells

Human colorectal cancer cells SW480 were grown in RPMI-1640 (PAA) containing 10% fetal calf serum (Biochrom), DLD1 and HCT116 cells were grown in DMEM (PAA) containing 10% fetal calf serum. The cells were cultured in a humidified 5% CO_2_ atmosphere. Cells were analyzed by PCR and found to be free of mycoplasms. Authentification of SW480, DLD1 and HCT116 cells was performed by short tandem repeat (STR) genotyping (Leibniz-Institut DSMZ, Braunschweig, Germany) STR genotype was consistent with published genotype. Cells were transfected with the plasmids pcDNA3.1/MACC1/wt, pcDNA3.1/MACC1/L31V, pcDNA3.1/MACC1/S515L, pcDNA3.1/MACC1/R804T by using Fugene HD (Roche) according to the manual.

### Protein extraction and Western blotting

For protein isolation cells were harvested and incubated on ice with RIPA buffer (50 mM Tris–HCl pH 7.5, 150 mM NaCl, 1% Nonidet P-40, protease inhibitor tablets; Roche). Protein concentration was quantified with Coomassie Plus (Bradford) Protein Assay (Pierce). Fifty μg of the lysates were loaded on a NuPAGE Bis-Tris Gel (Invitrogen), separated, and transferred onto a nitrocellulose membrane. Membranes were incubated in blocking solution and afterwards incubated with rabbit anti-human MACC1 (1:1.000, Sigma) or mouse anti-human β-tubulin (1:1.000, BD Pharmingen) antibody, and subsequently with HRP-conjugated anti-rabbit IgG (1:10.000, Promega) or goat anti-mouse IgM (1:10.000, Sigma) antibody, respectively. At least 3 independent experiments were performed.

### Migration, proliferation and wound healing assays

Cell migration assay of the cell lines SW480, DLD1, HCT116 and their cell clones was performed in transwell Boyden chambers (Invitrogen). Cells that passed the pores and migrated through the lower chamber were counted after 24 h. For the proliferation assays cells were treated daily with 3-(4,5-dimethyl-2-thiazol)-2,5-diphenyl-2 H-tetrazolium bromide (MTT; Sigma), crystallized MTT was resolved with dimethylsulfoxid (Applichem), and the optical density was measured at 560 nm. For the wound healing assays cells were seeded into wells, scratched, and the wound healing area was documented until day 4. Assays were repeated 3 times for each clone, respectively.

### Statistics

Statistical analysis was performed with GraphPad Prism version 5. The comparison of two groups was done by two-sided Student’s *t*-test. Several groups were compared by one-way analysis of variance (ANOVA) and Bonferroni post hoc multiple comparison. The significance of clinical parameters was evaluated by Mann–Whitney tests and Chi-Square-tests with one degree of freedom. The odds ratio (OR) or hazard ratio (HR) with corresponding 95% confidence intervals (CI) were calculated. The Kaplan–Meier method was used to estimate cumulative survival rates, and differences in survival rates were assessed using the Log-Rank test. The OEGE software was used for testing the Hardy-Weinberg equilibrium (HWE) using the Chi-Square-test with one degree of freedom [24]. For the determination of the linkage disequilibrium the correlation coefficient r^2^ and D’ was calculated by using OEGE software [25]. Statistical significance was considered for P < 0.05.

## Competing interests

The authors declare no conflict of interest.

## Authors' contributions

FS and US conceived and designed the experiments. FS and KK carried out the experimental work. SB and PMS provided clinical information. FS and SB analyzed the data and performed statistical analyses. FS and US wrote the paper. All authors read and approved the final manuscript.

## Supplementary Material

Additional file 1 ** Table S1.** Summary of tissues obtained from 154 colorectal carcinoma patients. Click here for file
