# Supplementary material for: SNPs in the coding region of the metastasis-inducing gene MACC1 and clinical outcome in colorectal cancer
Source: Mol Cancer. 2012 Jul 29;11:49. doi: 10.1186/1476-4598-11-49 (PMC3480947; doi:10.1186/1476-4598-11-49)
Supplement: Additional file 1 — Table S1. Summary of tissues obtained from 154 colorectal carcinoma patients. [file 1476-4598-11-49-S1.doc]

**Supplementary Table 1 – Summary of tissues obtained from 154 colorectal carcinoma patients**

| **Number** | **Sex** | **Age** | **Localisation** | **T** | **N** | **Meta-**  **chronous Metastasis** | **stages** | **cg (rs4721888)** | **ct (rs975263)** | **gc (rs3735615)** | **cc (rs3735615)** |
| --- | --- | --- | --- | --- | --- | --- | --- | --- | --- | --- | --- |
| 1 | m | 58 | colon | 3 | 0 |  | 2 |  |  | x | x |
| 2 | f | 51 | rectum | 3 | 1 |  | 3 |  |  | x |  |
| 3 | m | 60 | rectum | 3 | 0 |  | 2 |  |  | x |  |
| 4 | f | 62 | rectum | 2 | 1 |  | 3 |  |  | x |  |
| 5 | f | 67 | colon | 1 | 2 |  | 3 | x | x | x | x |
| 6 | f | 77 | colon | 3 | 1 |  | 3 |  |  | x |  |
| 7 | m | 62 | colon | 3 | 0 |  | 2 | x | x | x | x |
| 8 | m | 79 | colon | 3 | 1 |  | 3 |  | x | x | x |
| 9 | m | 64 | colon | 3 | 0 |  | 2 |  |  | x | x |
| 10 | m | 66 | colon | 3 | 0 |  | 2 |  | x | x |  |
| 11 | m | 61 | rectum | 3 | 0 |  | 2 |  |  | x | x |
| 12 | m | 62 | colon | 3 | 0 |  | 2 |  | x | x |  |
| 13 | m | 58 | colon | 3 | 0 |  | 2 |  |  | x | x |
| 14 | f | 70 | colon | 4 | 2 |  | 3 |  |  |  |  |
| 15 | f | 53 | colon | 3 | 0 |  | 2 |  | x | x | x |
| 16 | f | 71 | rectum | 3 | 0 |  | 2 |  |  |  |  |
| 17 | f | 44 | rectum | 3 | 0 |  | 2 |  |  | x | x |
| 18 | f | 64 | colon | 3 | 0 |  | 2 |  |  | x |  |
| 19 | m | 71 | colon | 3 | 1 |  | 3 |  |  | x |  |
| 20 | m | 64 | rectum | 1 | 0 |  | 1 | x | x | x | x |
| 21 | f | 73 | colon | 3 | 0 |  | 2 |  |  | x | x |
| 22 | f | 59 | colon | 2 | 0 |  | 1 |  |  | x |  |
| 23 | f | 48 | rectum | 3 | 1 | x | 3 |  | x | x |  |
| 24 | m | 60 | colon | 3 | 2 |  | 3 |  |  | x | x |
| 25 | f | 63 | colon | 3 | 0 |  | 2 |  |  | x |  |
| 26 | f | 69 | rectum | 2 | 0 |  | 1 |  |  | x |  |
| 27 | m | 77 | rectum | 2 | 0 | x | 1 |  |  | x | x |
| 28 | m | 71 | colon | 3 | 0 |  | 2 |  |  | x |  |
| 29 | f | 77 | rectum | 3 | 0 |  | 2 |  | x | x | x |
| 30 | m | 53 | colon | 3 | 0 |  | 2 | x | x | x | x |
| 31 | f | 63 | colon | 3 | 2 | x | 3 |  | x | x | x |
| 32 | m | 45 | colon | 3 | 0 |  | 2 | x | x | x |  |
| 33 | f | 58 | colon | 3 | 1 |  | 3 |  |  | x |  |
| 34 | f | 59 | colon | 3 | 1 |  | 3 | x | x | x | x |
| 35 | f | 57 | rectum | 2 | 1 |  | 3 |  | x | x |  |
| 36 | f | 88 | colon | 4 | 0 |  | 2 |  |  | x | x |
| 37 | m | 71 | rectum | 3 | 1 |  | 3 |  |  | x |  |
| 38 | m | 60 | rectum | 3 | 1 |  | 3 | x | x | x |  |
| 39 | m | 65 | rectum | 3 | 1 |  | 3 |  |  | x |  |
| 40 | f | 74 | colon | 2 | 0 |  | 1 |  |  |  |  |
| 41 | f | 64 | colon | 3 | 0 |  | 2 |  | x | x | x |
| 42 | f | 81 | colon | 1 | 0 |  | 3 |  |  | x |  |
| 43 | f | 73 | rectum | 3 | 0 |  | 2 |  |  | x |  |
| 44 | m | 59 | colon | 3 | 0 |  | 2 |  | x | x |  |
| 45 | f | 69 | rectum | 4 | 2 |  | 3 |  |  | x |  |
| 46 | f | 45 | colon | 3 | 0 |  | 2 |  |  |  |  |
| 47 | f | 53 | colon | 3 | 1 |  | 3 |  | x | x | x |
| 48 | f | 65 | colon | 3 | 0 |  | 2 |  |  | x |  |
| 49 | f | 80 | colon | 4 | 0 |  | 2 |  | x | x |  |
| 50 | m | 60 | colon | 3 | 0 |  | 2 |  | x | x | x |
| 51 | f | 67 | colon | 3 | 0 |  | 2 |  | x | x |  |
| 52 | m | 65 | colon | 3 | 2 |  | 3 |  |  | x | x |
| 53 | f | 58 | rectum | 2 | 2 |  | 3 |  |  | x | x |
| 54 | f | 51 | rectum | 3 | 2 |  | 3 |  |  | x | x |
| 55 | m | 60 | rectum | 1 | 0 |  | 1 |  |  | x | x |
| 56 | m | 62 | colon | 3 | 0 |  | 2 |  |  | x |  |
| 57 | m | 67 | colon | 3 | 1 |  | 3 |  | x | x |  |
| 58 | m | 77 | colon | 3 | 0 |  | 2 |  | x | x | x |
| 59 | m | 62 | colon | 3 | 3 |  | 3 |  |  | x |  |
| 60 | m | 79 | colon | 3 | 1 |  | 3 |  |  |  |  |
| 61 | m | 64 | rectum | 3 | 0 |  | 2 |  | x | x | x |
| 62 | f | 66 | colon | 3 | 0 |  | 2 |  |  | x | x |
| 63 | f | 61 | colon | 3 | 0 |  | 2 |  |  | x |  |
| 64 | f | 62 | colon | 4 | 1 | x | 3 |  | x | x |  |
| 65 | f | 58 | rectum | 3 | 0 |  | 2 | x | x | x |  |
| 66 | f | 70 | colon | 3 | 0 |  | 2 |  | x | x |  |
| 67 | f | 53 | colon | 3 | 0 |  | 2 |  |  |  |  |
| 68 | f | 71 | colon | 4 | 1 |  | 3 | x | x | x | x |
| 69 | m | 44 | colon | 3 | 0 |  | 2 |  | x | x | x |
| 70 | m | 64 | colon | 3 | 0 |  | 2 | x | x | x | x |
| 71 | m | 71 | colon | 3 | 1 |  | 3 |  | x | x |  |
| 72 | f | 64 | colon | 3 | 0 |  | 2 | x | x | x | x |
| 73 | m | 73 | colon | 3 | 0 |  | 2 |  | x |  |  |
| 74 | m | 59 | colon | 3 | 0 |  | 2 |  |  |  |  |
| 75 | m | 48 | rectum | 2 | 0 |  | 1 |  | x | x |  |
| 76 | m | 60 | rectum | 3 | 0 |  | 2 |  |  | x | x |
| 77 | f | 63 | colon | 3 | 1 |  | 3 |  |  |  |  |
| 78 | m | 69 | colon | 3 | 0 | x | 2 |  |  | x |  |
| 79 | m | 77 | rectum | 3 | 0 |  | 2 |  | x | x | x |
| 80 | m | 71 | colon | 1 | 0 |  | 1 |  | x | x | x |
| 81 | f | 77 | colon | 3 | 1 |  | 3 |  |  | x |  |
| 82 | m | 53 | colon | 3 | 0 |  | 2 |  | x | x |  |
| 83 | m | 63 | colon | 3 | 1 |  | 3 |  |  | x | x |
| 84 | f | 45 | colon | 4 | 0 |  | 2 |  |  |  |  |
| 85 | f | 58 | colon | 3 | 0 |  | 2 |  | x | x |  |
| 86 | f | 59 | rectum | 3 | 1 |  | 3 |  | x | x |  |
| 87 | m | 57 | colon | 3 | 0 |  | 2 |  | x | x |  |
| 88 | f | 88 | colon | 3 | 0 |  | 2 |  |  | x |  |
| 89 | f | 71 | rectum | 4 | 1 |  | 3 |  |  |  |  |
| 90 | f | 60 | colon | 2 | 0 |  | 1 |  |  | x |  |
| 91 | m | 65 | colon | 3 | 0 |  | 2 |  | x | x | x |
| 92 | m | 74 | colon | 3 | 0 |  | 2 |  |  |  |  |
| 93 | f | 64 | colon | 3 | 0 |  | 2 |  |  | x |  |
| 94 | m | 81 | rectum | 3 | 1 |  | 3 | x | x | x | x |
| 95 | f | 73 | rectum | 2 | 0 |  | 1 |  | x | x | x |
| 96 | m | 59 | rectum | 2 | 0 |  | 1 |  |  | x | x |
| 97 | m | 69 | colon | 2 | 0 | x | 1 |  |  | x |  |
| 98 | m | 45 | rectum | 2 | 0 |  | 1 |  | x | x |  |
| 99 | m | 53 | rectum | 2 | 0 |  | 1 |  |  | x |  |
| 100 | m | 65 | rectum | 2 | 0 |  | 1 |  |  |  |  |
| 101 | f | 80 | colon | 1 | 0 |  | 1 |  | x | x |  |
| 102 | m | 60 | rectum | 1 | 0 |  | 1 |  |  | x |  |
| 103 | f | 67 | rectum | 2 | 0 |  | 1 |  | x | x |  |
| 104 | m | 65 | rectum | 1 | 0 |  | 1 |  |  |  |  |
| 105 | m | 58 | colon | 2 | 0 |  | 1 | x | x | x |  |
| 106 | m | 51 | colon | 1 | 0 |  | 1 |  | x | x | x |
| 107 | m | 60 | rectum | 2 | 0 |  | 1 |  |  |  |  |
| 108 | f | 62 | rectum | 2 | 0 |  | 1 |  | x | x |  |
| 109 | m | 67 | colon | 1 | 0 |  | 1 |  |  | x |  |
| 110 | m | 77 | rectum | 2 | 0 | x | 1 |  | x | x |  |
| 111 | m | 62 | colon | 2 | 0 | x | 1 |  | x | x | x |
| 112 | m | 79 | colon | 3 | 0 |  | 2 | x | x | x |  |
| 113 | m | 64 | colon | 3 | 0 |  | 2 |  |  | x |  |
| 114 | f | 66 | colon | 3 | 0 |  | 2 | x | x | x | x |
| 115 | f | 61 | colon | 3 | 0 |  | 2 |  |  | x | x |
| 116 | f | 62 | colon | 3 | 0 |  | 2 |  | x |  |  |
| 117 | f | 58 | colon | 3 | 0 |  | 2 |  |  |  |  |
| 118 | f | 70 | colon | 3 | 0 |  | 2 |  | x | x | x |
| 119 | m | 53 | colon | 4 | 0 |  | 2 |  |  | x | x |
| 120 | f | 71 | colon | 4 | 0 |  | 2 |  |  |  |  |
| 121 | m | 44 | colon | 3 | 0 |  | 2 |  |  | x |  |
| 122 | m | 64 | colon | 3 | 0 |  | 2 |  |  | x |  |
| 123 | m | 71 | colon | 3 | 0 |  | 2 |  |  | x |  |
| 124 | f | 64 | colon | 3 | 0 | x | 2 |  | x | x |  |
| 125 | f | 73 | colon | 4 | 0 | x | 2 |  | x | x |  |
| 126 | m | 59 | rectum | 4 | 0 | x | 2 |  | x | x |  |
| 127 | f | 48 | rectum | 4 | 0 | x | 2 |  | x | x | x |
| 128 | m | 60 | rectum | 3 | 0 | x | 2 |  |  |  |  |
| 129 | m | 63 | colon | 3 | 0 | x | 2 |  |  | x | x |
| 130 | m | 69 | rectum | 4 | 0 | x | 2 |  | x | x | x |
| 131 | f | 77 | rectum | 4 | 0 | x | 2 |  |  | x | x |
| 132 | m | 71 | colon | 3 | 0 | x | 2 |  | x | x |  |
| 133 | m | 77 | rectum | 3 | 0 | x | 2 |  |  |  |  |
| 134 | f | 53 | colon | 3 | 0 | x | 2 |  |  | x |  |
| 135 | m | 63 | colon | 3 | 1 |  | 3 |  | x | x |  |
| 136 | m | 45 | colon | 3 | 1 |  | 3 |  | x | x | x |
| 137 | f | 58 | colon | 4 | 3 |  | 3 |  |  |  |  |
| 138 | m | 59 | colon | 3 | 3 |  | 3 | x | x | x | x |
| 139 | f | 57 | colon | 2 | 3 |  | 3 |  | x | x |  |
| 140 | f | 88 | colon | 3 | 1 |  | 3 | x | x | x |  |
| 141 | m | 71 | colon | 3 | 1 |  | 3 | x | x | x | x |
| 142 | m | 60 | colon | 1 | 1 |  | 3 |  | x | x |  |
| 143 | m | 65 | colon | 4 | 1 |  | 3 |  |  | x |  |
| 144 | m | 74 | rectum | 3 | 1 |  | 3 |  |  |  |  |
| 145 | m | 64 | rectum | 2 | 1 | x | 3 |  | x | x | x |
| 146 | f | 81 | colon | 2 | 1 | x | 3 | x | x | x |  |
| 147 | f | 73 | rectum | 3 | 1 | x | 3 |  | x | x | x |
| 148 | f | 59 | colon | 3 | 3 | x | 3 |  |  | x |  |
| 149 | m | 69 | colon | 3 | 1 | x | 3 | x | x | x |  |
| 150 | m | 45 | colon | 3 | 1 | x | 3 |  | x | x |  |
| 151 | f | 53 | colon | 2 | 1 | x | 3 |  | x | x |  |
| 152 | f | 65 | colon | 3 | 2 | x | 3 |  |  |  |  |
| 153 | m | 80 | rectum | 4 | 1 | x | 3 |  |  |  |  |
| 154 | m | 60 | colon | 3 | 1 | x | 3 |  |  |  |  |
